# Experimental Analysis of the Extensional Flow of Very Weakly Viscoelastic Polymer Solutions

**DOI:** 10.3390/ma13010192

**Published:** 2020-01-02

**Authors:** Manuel Rubio, Alberto Ponce-Torres, Emilio José Vega, José María Montanero

**Affiliations:** Depto. de Ingeniería Mecánica, Energética y de los Materiales and Instituto de Computación Científica Avanzada (ICCAEx), Universidad de Extremadura, E-06006 Badajoz, Spain; marubio@unex.es (M.R.); aponce@unex.es (A.P.-T.); ejvega@unex.es (E.J.V.)

**Keywords:** extencional flow, relaxation time, weakly viscoelastic solutions

## Abstract

We study with ultra-high-speed imaging the thinning of the filament formed during the breakup of a pendant droplet of very weakly viscoelastic polymer solutions of polyvinylpyrrolidone (PVP) and polyethylene oxide (PEO). In the latter case, we consider two molecular weights: $10^{5}$ g/mol (PEO100K) and $2\times 10^{6}$ g/mol (PEO2M). The results allow us to measure with high reproducibility extensional relaxation times of the order of 10 μs. Despite the noticeable differences between PVP and PEO100K, very similar values are obtained for the range of concentrations where the linear elasto-capillary is established. For PEO2M, the extensional relaxation time depends on the concentration even for values significantly smaller than the overlap one. The prediction $c_{\mathrm{low}}$ for the concentration below which the linear elasto-capillary regime cannot be reached qualitatively agrees with the results for PVP and PEO2M, while it underestimates the critical concentration for PEO100K. The results for PEO2M are consistent with those reported in the literature for higher concentrations.

## 1. Introduction

Weakly elastic polymer solutions have great relevance in many applications, such as inkjet printing and nanofiber spinning. For instance, flexible electronic circuits are fabricated with droplet-based inkjet printing of PEDOT:PSS (poly(3,4-ethylenedioxythiophene):poly(styrene sulfonate)) [1] due to its high electrical conductivity, transparency, physical and chemical stability, and capability to easily form films. PEDOT:PSS aqueous solutions exhibit a near-Newtonian character with densities and viscosities similar to those of water, which hinders the proper characterization of their viscoelasticity. The production of nanofibers and their mats is useful for a wide range of applications, including filters, membranes, microelectronics, military, optics or health, and personal care, among many others [2]. Membranes and scaffolds made up of nanofibers of low-molecular-weight PVP (polyvinylpirrolidone) are widely used for tissue engineering due to its biocompatibility and low toxicity [3]. It is well known that the fiber diameter can be greatly reduced by decreasing the polymer concentration. Experimental information about the extensional flow of polymer solutions with low molecular weights and low concentrations is very useful for the applications mentioned above and many others.

When a small amount of polymer with a moderate/high molecular weight is added to a Newtonian solvent, the solution dynamical behavior can experience substantial changes. One of those changes is the appearance of the elasto-capillary regime in the filament thinning taking place during the breakup of pendant drops, liquid bridges and jets [4]. In this regime, the presence of macromolecules makes the radius of a quasi-cylindrical thread decrease exponentially with time with a relaxation time $3\lambda_{e}$, where $\lambda_{e}$ is the so-called liquid extensional relaxation time [5,6]. The flow in the thread is essentially a uniaxial extensional flow characterized by the constant extensional rate $\dot{\varepsilon }=2/\left(3\lambda_{e}\right)$. Elastic stresses grow exponentially to balance the driving capillary pressure during the elasto-capillary thinning, while inertial, viscous and gravitational effects are negligible.

Filament stretching extensional rheometry (FiSER) [7] and capillary breakup extensional rheometry (CaBER) [8] are probably the most popular techniques to measure the extensional relaxation time $\lambda_{e}$ of a viscoelastic liquid. Most extensional rheometers operate appropriately only for sufficiently high viscosities $\eta_{0}$ and large enough values of $\lambda_{e}$. There have been notable efforts to reduce the lower operational limits of these two quantities. Among them, we can mention the Rayleigh-Ohnesorge jet elongational rheometer (ROJER) [9], the optically-detected elastocapillary self-thinning dripping-onto-substrate (ODES-DoS) method [10], the dripping-onto-substrate version of CaBER rheometry (CaBER-DoS) based on high-speed imaging [11], the slow retraction method (SRM) [12], and the fast capillary thinning extensional rheometer “Cambridge Trimaster” (CTM) [13]. Extensional relaxation times down to values as small as 60 μs have been measured with ROJER [14]. These values may have to be multiplied by a factor 3/2 because the jet thinning follows a scaling different from that of a liquid bridge [15]. ODES-DoS is capable of measuring relatively small relaxation times ($\lambda_{e}≲1$ ms) of low-viscosity ($\eta_{0}≲20$ mPa s) liquids. CaBER-DoS has been recently used to determine relaxation times of the order of 100 μs [11]. Relaxation times as low as 80 μs have been measured with the CTM device [13]. Using the SRM method, relaxation times around 240 μs were obtained for aqueous solutions of polyethylene oxide (PEO) [12]. When the liquid bridge was surrounded by an oil bath, solvent evaporation was eliminated, and extensional relaxation times just above 100 μs were accurately measured with the SRM method [16]. The extensional relaxation time of weakly-elastic PEO solutions has been estimated from the filament radius for which the inertial regime gives rise to the elasto-capillary one during the breakup of a drop deposited on a substrate [11]. Extensional relaxation times of the order of or smaller than 10 μs have not been measured so far, which prevents the characterization of polymer solutions with low molecular weights and low concentrations [17,18].

One expects that, for polymer concentrations smaller than the coil overlap concentration $c^{*}$, the solution behaves as an ideal system, i.e., without direct or fluid-mediated interactions between polymers. In this case, the stress relaxation time must become independent from the polymer concentration. However, capillary thinning measurements show that the effective relaxation time $\lambda_{e}$ characterizing the transient uniaxial extensional flow strongly depends on the polymer concentration even at concentrations well below the coil overlap concentration $c^{*}$. This deviation with respect to both theoretical predictions and small amplitude oscillatory shear (SAOS) measurements of the relaxation time has been explained in terms of the influence of the underlying solvent viscous stress and the finite extensibility of the chains [19]. Specifically, the combination of these two effects prevents weakly elastic fluids from reaching the proper elastocapillary regime required for the correct determination of the relaxation time. For this reason, spurious concentration dependence of the relaxation time may be obtained in capillary thinning experiments with ultra-dilute polymer solutions. In addition, it has been shown that polymer chains may interact at concentrations significantly below $c^{*}$, even under equilibrium conditions [20]. The extensional flow of polymer solutions with low-molecular weights at concentrations smaller than $c^{*}$ has not been tested due to the smallness of the relaxation time. The present results provide useful experimental data under these conditions.

When the polymer concentration *c* is of the order of $c^{*}$, the relaxation time $\lambda_{e}$ scales as [21]

(1) $$\lambda_{e}∼c^{\left(2-3\nu )/(3\nu -1\right)},$$

where ν is the exponent indicating the quality of the solvent. If the polymer concentration *c* is well below $c^{*}$ (typically, $c/c^{*}≲10^{-1}$), the relaxation time $\lambda_{e}$ is expected to be approximately equal to the longest Zimm relaxation time [22]

(2) $$\lambda_{Z}=\frac{F\left[\eta \right]M_{w}\eta_{s}}{R_{g}T},$$

where $F=1/(\sum_{i=1}^{N}\left(1/i^{3\nu }\right)$, [η] is the intrinsic viscosity, $M_{w}$ the molecular weight, $\eta_{s}$ the solvent viscosity, $R_{g}$ the gas constant, and *T* the temperature. One can calculate $\lambda_{Z}$ from experimental values of ν, [η] and $\eta_{s}$. The value of ν can be obtained by fitting (1) to experimental values of $\lambda_{e}$ for $c∼c^{*}$.

It is known that the strain rate $\dot{\varepsilon }$ before the transition from inertio-capillary to elasto-capillary regime increases well beyond $2/(3\lambda_{e})$ followed by a sudden drop to this value once the elasto-capillary regime is established [23]. If one assumes that the transition from inertio-capillary to elasto-capillary flow takes place when $\dot{\varepsilon }=2/\left(3\lambda_{e}\right)$, then the filament radius at that transition is [11]

(3) $$R^{*}≃{\left(\lambda_{e}\sigma /\rho \right)}^{1/2},$$

where σ and ρ are the surface tension and density, respectively. The development of ultra-fast imaging acquisition systems allows experimenters to observe the thinning of viscoelastic filaments with increasing spatio-temporal resolutions. Tens of images can be acquired during this process for filament radius smaller than $R^{*}∼10$μm [24]. According to the estimate (3), this leads to potentially measurable values of $\lambda_{e}$ down to a few microseconds, which opens the door to the characterization of very weakly-elastic fluids.

In this paper, we implement an experimental technique capable of measuring extensional relaxation times of the order of 10 μs. The method is based on the capillary thinning of the filament arising during the breakup of a pendant droplet hanging on a submillimeter feeding capillary. The results for polyvinylpyrrolidone (PVP) and PEO solutions show that a proper elasto-capillary regime emerges right before the breakup, from which the extensional relaxation time can be accurately determined. The method provides values of $\lambda_{e}$ with high reproducibility.

## 2. Materials and Methods

**Figure 1:**
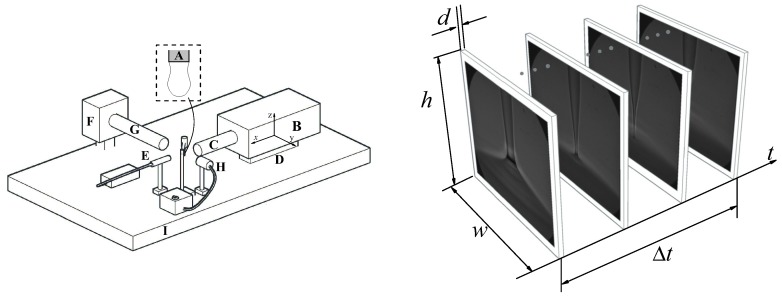
(**Left**) Experimental setup: feeding capillary (A), ultra-high speed video camera (B), optical lenses (C), triaxial translation stage (D), laser (E), optical trigger (F), optical lenses (G), optical fiber (H), and anti-vibration isolation system (I). (**Right**) Minimum spatio-temporal hypervolume analyzed in the experiment: image width w=94μm, height h=78μm, depth of field d=0.48μm and time Δt=36 μs elapsed during the experiment.

In the experimental setup (Figure 1), a cylindrical feeding capillary (A) $R_{0}=115$μm in outer radius is placed vertically. A pendant droplet is formed by injecting the liquid at a constant flow rate with a syringe pump (Harvard Apparatus PHD 4400, Holliston, MA, USA) connected to a stepping motor. We used a high-precision orientation system and a translation stage to ensure the correct position and alignment of the needle. Digital images of the drop were taken using an ultra-high-speed video camera (KIRANA-5, SPECIALISED IMAGING, Pitstone, UK) (B) equipped with optical lenses (an Optem HR 50× magnification zoom-objective and a NAVITAR 12X set of lenses) (C). As will be explained below, the images were acquired either at 5× 10${}^{6}$ fps with a magnification 101.7 nm/pixel or at 5 × 10${}^{5}$ fps with a magnification 156 nm/pixel. The camera could be displaced both horizontally and vertically using a triaxial translation stage (D) with one of its horizontal axes (axis *x*) motorized (THORLABS Z825B) and controlled by the computer, which allowed us to set the droplet-to-camera distance with an error smaller than 29 nm. The camera was illuminated with a laser (SI-LUX 640, Specialised Imaging) (E) synchronized with the camera, which reduced the effective exposure time down to 100 ns. The camera was triggered by an optical trigger (SI-OT3, Specialised Imaging) ) (F) equipped with optical lenses (G) and illuminated with cold white backlight provided by an optical fiber (H). All these elements were mounted on an optical table with a pneumatic anti-vibration isolation system (I) to damp the vibrations coming from the building.

In the experiment, a pendant droplet hanging on the feeding capillary was inflated by injecting the liquid at the flow rate 1 mL/h. The triple contact lines anchored to the outer edge of the capillary. The drop reached its maximum volume stability limit after around 20 s. When the maximum volume stability limit was reached, the droplet broke up spontaneously. We recorded 180 images of the final stage of the breakup process at rates up to 5 × 10${}^{6}$ fps within a frame of down to 94 × 78 μm. This experiment was repeated several times to assess the degree of reproducibility of the experimental results. The flow rate at which the pendant droplet is inflated was reduced down to 0.1 mL/h to verify that this parameter does not affect the final stage of the breakup process. Besides, 180 images of a frame of 144 × 120 μm were taken at 5 × 10${}^{5}$ fps to describe the process on a larger scale. We processed the images acquired in the experiments to calculate the radius $R_{c}$ of the central part of the filament as a function of time. As will be explained in Section 3, the effect on $R_{c}$ of the filament section used to determine that radius is negligible.

**Figure 2:**
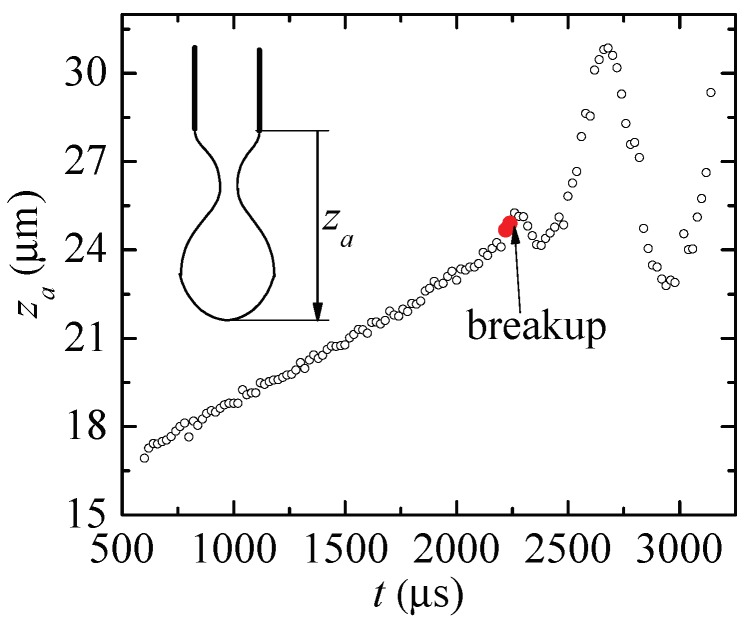
Vertical position $z_{a}$ of the apex during the pendant drop breakup for PEO ($M_{w}=2\times 10^{6}$ g/mol, *c* = 0.0003%). The red big circles indicate the time interval used to determine the relaxation time.

The idea of any capillary breakup extensional rheometer is to produce a filament shrinkage driven by the capillary instability in the hope that an elasto-capillary regime is reached, where the radius of a quasi-cylindrical thread decreases exponentially under the action of surface tension and polymer stresses. This can be done with several fluid configurations such as liquid bridges, jets and pendant drops. As discussed by [25], pendant drops can be used to measure the relaxation time independently of whether the droplet touches the substrate or not. Relevant examples of the use of pendant drops for this purpose were given by [23,26]. One may still wonder whether the apex displacement taking place during the filament stretching, not present in the DoS technique, can affect the measurement of the extensional relaxation time. Figure 2 shows the vertical position $z_{a}$ of the apex during the pendant drop breakup for one of the aqueous solutions of PEO considered in this work. The two red big circles indicate the tiny time interval used to determine the relaxation time. During that interval, the droplet apex moved a distance less than 0.3% the length of the stretching filament. The apex position oscillation results from the capillary waves triggered by the pinch-off of the free surface.

The test fluids used in the experiments were polymeric solutions in deionized water (DIW) of PVP (Sigma Aldrich, $M_{w}=3.6\times 10^{5}$ g/mol) and PEO with two molecular weights: PEO100K (Sigma Aldrich, $M_{w}=10^{5}$ g/mol, PEO100K) and PEO2M (Sigma Aldrich, $M_{w}=2\times 10^{6}$ g/mol). Stock solutions with several concentrations (wt %) were prepared by dissolving the polymers in the solvent with a magnetic stirrer at low angular speeds to minimize mechanical degradation of the long polymer chains. The PVP concentrations were 0.07% (PVP0.07), 0.1% (PVP0.1), 0.2% (PVP0.2), 0.4% (PVP0.4), 0.6% (PVP0.6), 1% (PVP1), 2% (PVP2) and 3% (PVP3). The PEO100K concentrations were 0.01% (PEO100K-0.01), 0.05% (PEO100K-0.05), 0.1% (PEO100K-0.1), 0.2% (PEO100K-0.2), 0.5% (PEO100K-0.5), 1% (PEO100K-1), 2% (PEO100K-2) and 5% (PEO100K-5). The PEO2M concentrations were 0.0003% (PEO2M-0.0003), 0.0005% (PEO2M-0.0005), 0.001% (PEO2M-0.001), 0.002% (PEO2M-0.002), 0.004% (PEO2M-0.004), 0.007% (PEO2M-0.007), 0.01% (PEO2M-0.01), 0.03% (PEO2M-0.03) and 0.1% (PEO2M-0.1).

We calculated the values of the parameters that may affect the filament thinning. The overlap concentration $c^{*}$ can be calculated as [19]

(4) $$c^{*}=\frac{M_{w}}{4/3\;\pi R_{G}^{3}N_{A}},$$

where $R_{G}$ is the gyration radius and $N_{A}$ is the Avogadro’s number. The swelling ratio $k_{\alpha }$ can be obtained from the formula $k_{\alpha }^{2}=F\left(1.5\right)/F\left(3\nu \right)$ [12]. The finite extensibility parameter $L^{2}$ can be estimated as [19]

(5) $$L^{2}=\frac{3}{k_{\alpha }^{2}}{\left[\frac{j\sin^{2}\left(\theta /2\right)M_{w}}{C_{\infty }M_{u}}\right]}^{2(1-\nu )},$$

where *j*, θ, and $M_{u}$ are the number of bonds, C-C bond angle and molar mass of the monomer, respectively, while $C_{\infty }$ is the characteristic ratio. The concentration threshold above which a true elasto-capillary regime appears has been estimated as [12]

(6) $$c_{\mathrm{low}}=\frac{M_{w}}{2.46R_{g}TL^{3/2}}{\left(\frac{\sigma^{2}\rho }{\lambda_{Z}^{2}}\right)}^{1/3}.$$

The minimum concentration to observe an elastic contribution during the droplet breakup can be calculated as [19]

(7) $$c_{\min}=\frac{3}{2}\frac{M_{w}\eta_{s}}{R_{g}T\lambda_{Z}L^{2}}.$$

Table 1 shows the properties of the three polymeric solutions analyzed in this paper.

## 3. Results and Discussion

**Figure 3:**
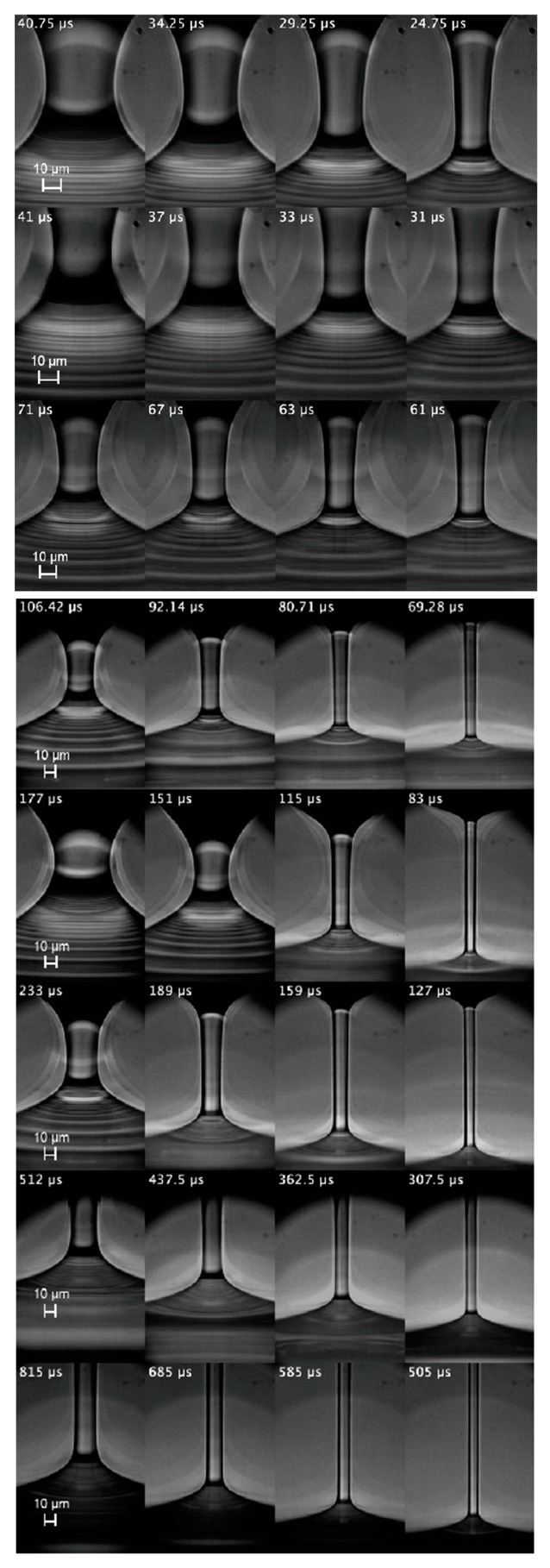
(From top to bottom) Images of the filament thinning over the elasto-capillary regime of PVP0.07, PVP0.1, PVP0.2, PVP0.4, PVP0.6, PVP1, PVP2 and PVP3. The labels indicate the time to the pinching. The white line separates the discarded realizations from those were the linear elasto-capillary regime was reached.

**Figure 4:**
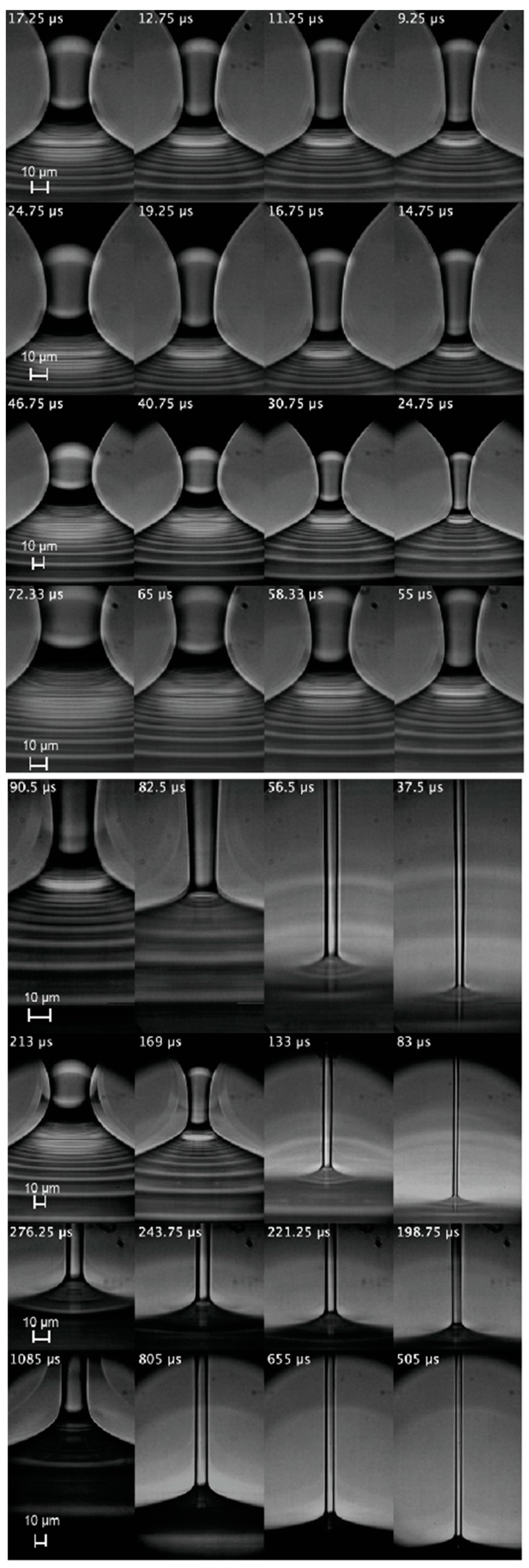
(From top to bottom) Images of the filament thinning over the elasto-capillary regime of PEO100K-0.01, PEO100K-0.05, PEO100K-0.1, PEO100K-0.2, PEO100K-0.5, PE100K-O1, PEO100K-2 and PEO100K-5. The labels indicate the time to the pinching. The white line separates the discarded realizations from those were the linear elasto-capillary regime was reached.

**Figure 5:**
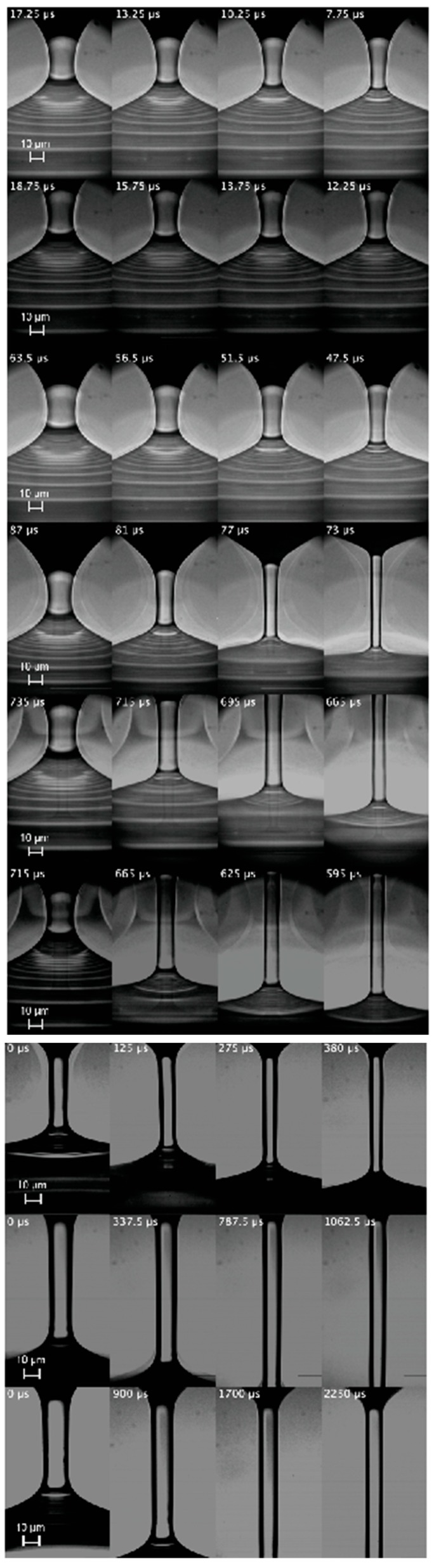
(From top to bottom) Images of the filament thinning over the elasto-capillary regime of PEO2M-0.0003, PEO2M-0.0005, PEO2M-0.001, PEO2M-0.002, PEO2M-0.004, PEO2M-0.007, PEO2M-0.01, PEO2M-0.03 and PEO2M-0.1. The labels indicate the time to the pinching. The white line separates the discarded realizations from those were the linear elasto-capillary regime was reached.

To measure the extensional relaxation time, we selected those time intervals that verify two conditions: (i) $R_{c}\left(t\right)$ deviated in less than 5% from the minimum radius of the filament, and (ii) $R_{c}\left(t\right)$ followed the exponential decay

(8) $$R_{c}\left(t\right)=A\exp\left(-\frac{t}{3\lambda_{e}}\right)=B\exp\left(\frac{\tau }{3\lambda_{e}}\right)$$

for a time interval larger than $\lambda_{e}$. Here, τ is the time to the pinching. As will be explained below, these geometric and kinematic requisites must be regarded as necessary but not sufficient conditions to determine the extensional relaxation time of a true elasto-capillary regime. Figure 3, Figure 4 and Figure 5 show images of the filament thinning over the selected time intervals for PVP, PEO100K and PEO2M, respectively.

As mentioned in Section 3, the height in the images used to determine $R_{c}\left(t\right)$ was chosen arbitrarily within the central part of the filament. To test that this choice does not significantly affect the value of the extensional relaxation time $\lambda_{e}$, we repeated the analysis considering different heights in the central part of the filament. Specifically, we increased and decreased the height used to determine $R_{c}\left(t\right)$ by a distance $0.5D_{neck}$, where $D_{neck}$ is the filament neck diameter in the first image of the sequences shown in Figure 3, Figure 4 and Figure 5. The variation of the extensional relaxation time was smaller than 10%.

**Figure 6:**
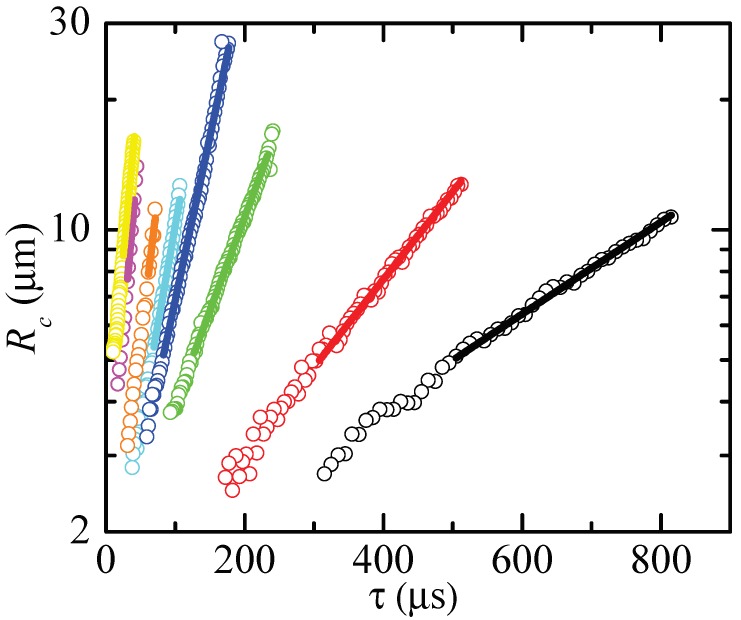
$R_{c}$ as a function of the time to pinching τ for PVP0.07 (yellow), PVP0.1 (magenta), PVP0.2 (orange), PVP0.4 (cyan), PVP0.6 (blue), PVP1 (green), PVP2 (red) and PVP3 (black). The solid lines are the fits of the function (8) to the experimental data.

**Figure 7:**
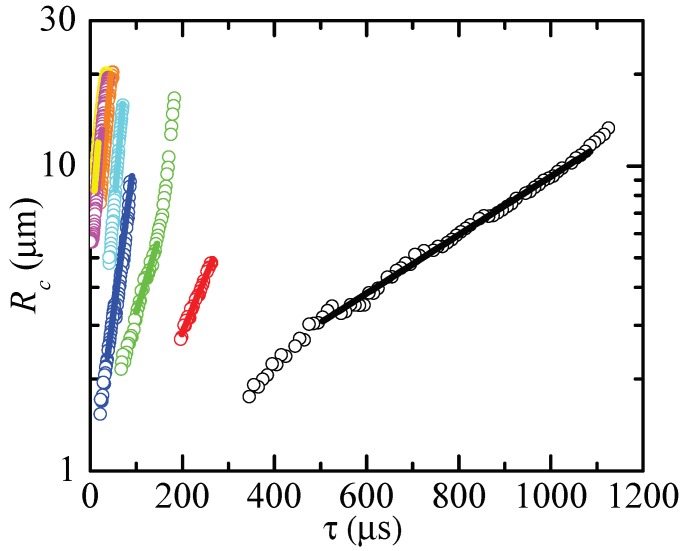
$R_{c}$ as a function of the time to pinching τ for PEO100K-0.01 (yellow), PEO100K-0.05 (magenta), PEO100K-0.1 (orange), PEO100K-0.2 (cyan), PEO100K-0.5 (blue), PEO100K-1 (green), PEO100K-2 (red) and PEO100K-5 (black). The solid lines are the fits of the function (8) to the experimental data.

**Figure 8:**
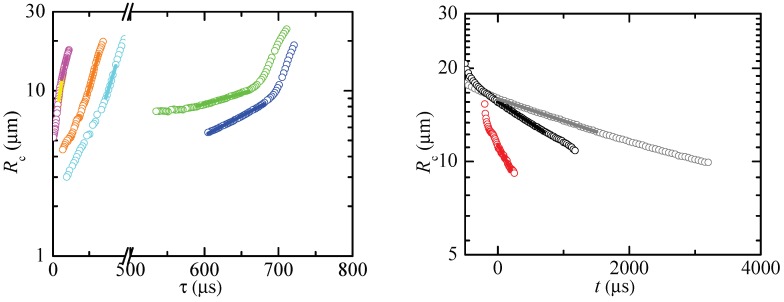
$R_{c}$ as a function of the time to pinching τ (**left**) and time *t* (**right**) for PEO2M-0.0003 (yellow), PEO2M-0.0005 (magenta), PEO2M-0.001 (orange), PEO2M-0.002 (cyan), PEO2M-0.004 (blue), PEO2M-0.007 (green), PEO2M-0.01 (red), PEO2M-0.03 (black) and PEO2M-0.1 (gray). The solid lines are the fits of the function (8) to the experimental data.

Figure 6, Figure 7 and Figure 8 show the values of $R_{c}$ and the corresponding fits (8). The time to the pinching τ is the natural temporal coordinate in most of the cases analyzed [26]. For the larger relaxation times, we used the time *t* measured from the first instant of the fitting (Figure 8-right). As can be seen, the radius approximately followed the exponential law (8) even for the lowest concentrations.

The characteristic time $\lambda_{e}$ corresponds to the true extensional relaxation time only if two additional conditions are verified: (i) the capillary pressure is balanced by elastic stresses (inertia and solvent viscosity are negligible) and (ii) the stretching polymer chains have not left their linear response regime over the analyzed time interval. To determine whether these conditions are verified in the selected time intervals, we consider the last image of the sequence, and compare the driving capillary pressure $p_{c}=\sigma /\left(2R_{c}\right)$ [12] with the upper bound $\tau_{e}=0.1GL^{2}$ of the elastic stress in the linear regime [32], where $G=cN_{A}k_{B}T/M_{w}$ is the modulus and $k_{B}$ the Boltzmann constant. This comparison leads to three scenarios:

- If $p_{c}\gg \tau_{e}$ then there are two possibilities: (i) the capillary pressure is balanced by inertia or solvent viscosity, and (ii) the elastic stress is much larger than the upper bound $\tau_{e}$ of the linear regime, and, therefore, the linearity condition does not hold.
- If $p_{c}\ll \tau_{e}$ then the elastic stress is much smaller than $\tau_{e}$, which suggests that the polymer stretching has hardly taken place in the preceding sequence of images, and inertia and/or solvent viscosity are expected to play a significant role.
- If $p_{c}∼\tau_{e}$ then we expect that capillary pressure is balanced by the elastic stress in the linear regime. Therefore, the analyzed sequence of images is assumed to correspond to the linear elasto-capillary regime.

The condition $p_{c}$∼$\tau_{e}$ for the last image of the selected sequence is simple but not the only criterion to discriminate between apparent and true elasto-capillary regimes. Other choices may also be appropriate. This discrimination is not usually conducted for viscoelastic solutions with polymers at higher concentrations or with larger molecular weights because it is implicitly assumed that the exponential decay of the radius of a quasi-cylindrical filament corresponds to the elasto-capillary regime.

The horizontal white lines in Figure 3, Figure 4 and Figure 5 separate the sequences of images for which $p_{c}∼\tau_{e}$ (the lower ones) from those for which this condition does not hold (the upper ones). For PVP, the first three concentrations were ruled out because $p_{c}\gg \tau_{e}$ in those cases. In fact, $\tau_{e}$ is much smaller than inertia $1/2\;\rho w^{2}$ in those experimental realizations. In this expression, the characteristic axial velocity can be estimated as $w=\dot{\varepsilon }\;L/2$, where $\dot{\varepsilon }=2/\left(3\lambda_{e}\right)$ and *L* are the strain rate and filament length, respectively. For PEO100K, the first four concentrations were ruled out because $p_{c}\ll \tau_{e}$ in those cases, which suggests that the true elastic stress is much smaller than $\tau_{e}$, and, therefore, elastic stresses hardly grew within the time interval considered. In fact, the capillary pressure was commensurate with inertia in those experimental realizations, as expected in the initial stage of the droplet breakup. For PEO2M, the first six concentrations were ruled out because $p_{c}\gg \tau_{e}$ in those cases. This probably occurs because the true elastic stress far exceeds the limit $\tau_{e}$ for the linear regime. In fact, inertia was subdominant as compared with the capillary pressure in those experimental realizations, which indicates that the capillary pressure was balanced by elastic stresses. Blistering was observed in these experiments for later times relatively close to the analyzed interval, which is another indicator that polymers left the linear regime in that interval. Finally, it should be noted that solvent viscous stresses were subdominant in practically all the experimental realizations. These stresses were estimated as $\tau_{s}=3\eta_{s}\dot{\varepsilon }$ with $\dot{\varepsilon }=2/\left(3\lambda_{e}\right)$.

The nozzle size plays an important role in the present experimental technique. For concentrations smaller than 0.1%, the Ohnesorge number Oh $=\eta_{s}/{\left(\rho \sigma R_{c}\right)}^{1/2}$ defined in terms of the filament radius $R_{c}$ takes values smaller than 0.1 even for $R_{c}<2$μm. This confirms that the solvent viscosity is subdominant over the droplet breakup, and elasticity competes with inertia to establish the sought elasto-capillary regime [12]. Under these circumstances, the Deborah number De $=\lambda_{e}{\left(\rho R^{3}/\sigma \right)}^{-1/2}$ defined in terms of the nozzle radius *R* essentially determines whether the elasto-capillary regime truly arises in the final stage of the breakup or, on the contrary, that thread evolution is contaminated by inertia effects all the way to the free surface pinch-off [25]. More precisely, the Deborah number must take sufficiently large values for the crossover between the inertio-capillary regime and the elasto-capillary balance to take place. The extensional relaxation time characterizing the smaller polymer concentrations takes values around 10 μs, which leads to Deborah numbers of the order of 0.1 for R=115μm. This means that elasto-capillary thinning is expected to be somewhat affected by inertia for the ultra-dilute polymer solutions. In fact, the images show the appearance of a mild neck in the region connecting the thread and the lower parent drop. In the experiments with PEO100K, one also observes the overturning of the free surface right before the breakup, a phenomenon characteristic of low-viscosity Newtonian pinch-off.

If the radius of the feeding capillary is increased, the capillary time increases, the Deborah number decreases, and inertia effects become more noticeable. Wagner et al. [26] pointed out the fact that the feeding capillary must be sufficiently small. Therefore, the use of larger feeding capillaries reduces the capacity of this technique to measure accurately relaxation times of ultra-dilute polymer solutions. We have measured the relaxation times of the PEO100K and PEO2M polymer solutions using a feeding capillary R=230μm in radius. None of the experiments with PEO100K verified the criterium $p_{c}∼\tau_{e}$. In fact, the images show the formation of thin necks sharper than their counterparts for R=115μm. Those images also show a more pronounced free surface overturning. In principle, the reduction of the feeding capillary size produces the desired effect of increasing the Deborah number. However, this reduction demands even higher spatio-temporal resolutions to visualize the last stage of the filament thinning.

**Figure 9:**
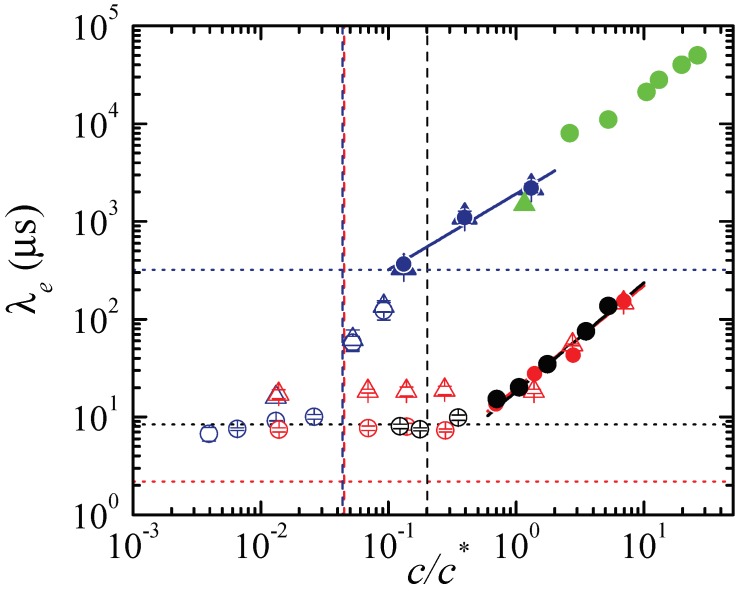
Mean values of the measured extensional relaxation times $\lambda_{e}$ as a function of the polymer reduced concentration $c/c^{*}$ for PVP (black symbols), PEO100K (red symbols) and PEO2M (blue symbols), respectively. The solid and open symbols correspond to true and discarded values, respectively. The circles and triangles correspond to the results measured with R=115 and 205 μm, respectively. The error bars indicate the standard deviations. For most of the solid symbols, these bars are not appreciated because they are smaller than the symbol size. The black, red and blue solid lines are the fits (1) to the experimental data within the interval $c/c^{*}>0.6$ for PVP and PEO100K and $c/c^{*}>0.1$ for PEO2M. The black, red and blue horizontal dotted lines indicate the longest Zimm relaxation time (2) for PVP, PEO100K, and PEO2M respectively. The black, red and blue vertical dashed lines indicate the values of $c_{\mathrm{low}}$ for PVP, PEO100K and PEO2M, respectively (the red and blue lines practically overlap). The green triangles and circles are data for PEO2M obtained from Refs. [17,18], respectively.

All the experiments were repeated 4 times for each liquid. The mean values of the measured extensional relaxation times are plotted in Figure 9 as a function of the polymer concentration. The standard deviations were smaller than 13.5% of the corresponding mean values. The average standard deviation was 4.5%, which shows the high reproducibility of the measurements. The figure shows the results for all the cases in which we observed the exponential thinning of a quasi-cylindrical filament. The solid symbols correspond to the cases that verify the additional criterium $p_{c}∼\tau_{e}$, while the open symbols are the cases ruled out. Extensional relaxation times of around 10 μs were measured, which is one order of magnitude smaller than the smallest relaxation times reported in the literature so far. Despite the noticeable differences between PVP and PEO100K, very similar values were obtained for the range of concentrations where the linear elasto-capillary regime was established. The extensional relaxation times for PEO100K and PEO2M differ by a factor around 50, which is consistent with the difference between their molecular weights. For PEO2M, the extensional relaxation time depended on the concentration even for values significantly smaller than the overlap one. The results obtained for the larger concentrations of PEO2M (those that verify $p_{c}∼\tau_{e}$) are practically the same for the two nozzles. As anticipated above, the linear elasto-capillary regime could not be found in any of the experiments for PEO100K with the larger nozzle. The relaxation times measured for the larger concentrations of PEO100K with the two nozzles practically coincide even though those obtained with the larger nozzle are not considered as true extensional relaxation times. The discarded values for PEO2M sharply decrease for $c/c^{*}≲0.05$ probably due to the acceleration of the filament thinning associated with finite extensibility effects. The values of $c_{\mathrm{low}}$ for PVP and PEO2M are good estimates for the concentrations below which the linear elasto-capillary regime was not reached. This critical concentration seems to be underestimated by $c_{\mathrm{low}}$ for PEO100K. Finally, Figure 9 also shows results for PEO2M reported in Refs. [17,18] for concentrations higher than those analyzed in the present work. As can be observed, our measurements are consistent with those results.

## 4. Conclusions

In this paper, we have presented an experimental technique for measuring extensional relaxation times of the order of 10 μs. The technique relies on the capillary thinning of the filament formed during the breakup of a pendant droplet hanging on a submillimeter feeding capillary. The method is not conceptually new. In fact, it is similar to CaBER-DoS, the major difference is the fact that very small droplets are broken before they are deposited on the substrate in our procedure. The method has been applied to characterize weakly-elastic solutions of PVP and PEO. An elasto-capillary regime arose right before the free surface pinching. We have verified that the results are highly reproducible, leading to extensional relaxation times with very small standard deviations.

The selected time intervals for the smaller concentrations did not verify the criterium $p_{c}∼\tau_{e}$ for the linear elasto-capillary regime. This does not necessarily mean that this regime did not appear during the breakup of the droplet. It probably arose during a short time, which does not allow the fitting of the exponential law (8) under the conditions established in this work.

## Figures and Tables

**Table 1:** Properties of the three polymeric solutions analyzed in this paper: molecular weight $M_{w}$, molar mass of the monomer $M_{u}$, overlap concentration $c^{*}$, solvent quality exponent ν, intrinsic viscosity [η] [23,27,28], gyration radius $R_{G}$ [27,28,29], longest Zimm relaxation time $\lambda_{Z}$, characteristic ratio $C_{\infty }$ [30], swelling ratio *k* [12], C-C bond angle of the monomer θ [30,31], number of bonds of the monomer *j* [30,31], finite extensibility parameter $L^{2}$ [19], concentration $c_{\mathrm{low}}$ above a true elasto-capillary regime appears [12], and minimum concentration $c_{\min}$ to observe an elastic contribution during the breakup [19].

Liquid	$M_{w}$ (g/mol)	$M_{u}$ (g/mol)	$c^{*}$ (wt %)	ν	[η] (mL/g)	$R_{G}$ (nm)	$\lambda_{Z}$ (μs)	$C_{\infty }$	$k_{\alpha }^{2}$	θ (${}^{\circ }$)	*j*	$L^{2}$	$c_{\mathrm{low}}$ (wt %)	$c_{\min}$ (wt %)
PVP	$3.6\times 10^{5}$	111.14	0.57	0.491	140.85	29.3	8.39	12.3	1	109.4	2	1169	0.114	0.00225
PEO100K	$10^{5}$	44.05	0.72	0.496	128.04	17.7	2.18	3.8	1	109.4	3	3790	0.032	0.00074
PEO2M	$2\times 10^{6}$	44.05	0.076	0.521	847.43	101.38	320.20	3.8	0.94	109.4	3	50104	0.0033	$7.67\times 10^{-6}$
